# A dynamic causal model on self-regulation of aversive emotion

**DOI:** 10.1186/s40708-020-00122-0

**Published:** 2020-12-09

**Authors:** Yang Yang, Xiaofei Zhang, Yue Peng, Jie Bai, Xiuya Lei

**Affiliations:** 1 Department of Psychology, Beijing Forestry University, Beijing, China; 2 Faculty of Information Technology, Beijing University of Technology, Beijing, China; 3 Department of Computer, Jiangsu University of Science and Technology, Zhenjiang, China

**Keywords:** Emotion regulation, Self-recovery, Dynamic causal modeling, fMRI

## Abstract

Cognitive regulation of emotion has been proven to be effective to take control the emotional responses. Some cognitive models have also been proposed to explain the neural mechanism that underlies this process. However, some characteristics of the models are still unclear, such as whether the cognitive regulation will be spontaneously employed by participants implicitly. The present study recruited the fMRI experiment to focus on the discomfort induced by viewing aversive pictures, and the emotional self-regulation during picture viewing. By using the dynamic causal modeling (DCM), 50 putative models of brain functional networks were constructed, one optimal model that fitted the real data best won the comparison from the candidates. As a result, the optimal model suggests that both the ventral striatum (VS)-centric bottom-up and the dorsolateral prefrontal cortex (DLPFC)-centric top-down regulations are recruited for self-regulation on negative emotions. The DLPFC will exert modulatory influence on the VS only when the VS fails to suppress the induced emotions by self-inhibition.

## Introduction

Emotions are generally recognized as responses generated by the human organism, which are essential for the adaptation to changing environmental demands [1–3]. Considering the significance, plenty of studies have been yielded in the recent years, focusing on emotional responses or experiences with the help of fMRI [4–6]. More neuroimaging studies concentrated on the emotions with negative valence induced by aversive stimuli, such as fear and disgust, to uncover the neural correlates underlying various psychiatric diseases, such as the obsessive–compulsive disorder (OCD), anxiety, and depressive disorders [7–11].

Much of the progress in studies of aversive emotions were closely associated with two research topics, one is the emotion formation and learning procedure, especially with the emotion conditioning based on the classic Pavlovian paradigm [12, 13]; the other topic is increasingly concerned in the recent two decades, which is the emotion regulation, i.e., the ability to exert control over one’s own emotional state [14–16]. It is a research subject related to the emotional post-processing, referring to operations that amplify, attenuate, or maintain an emotion [17, 18]. The dysfunction in emotion regulation has been argued to be crucial to account for the emotion-related diseases [19–22]. Therefore, individuals vulnerable to impaired regulation of aversive emotions are at risk for the maladaptation to the environment.

One important dimension of emotion regulation is the discrepancy between conscious (explicit) regulation and automatic (implicit) regulation. The former indicates cognitive manipulations guided by explicit intentions and accessible to one’s own awareness, while the latter conveys mental activities guided by implicit intentions or outside one’s awareness [23–26]. Although both forms of regulation are critical, relatively fewer neuroscience-based studies addressed the latter issue [27]. That is, studies have shown that when participants are exposed to experimental stimuli for arousing corresponding emotions (e.g., a crying woman in a picture for awakening sympathy), and instructed by specific task requirements (e.g., “imagine that the crying woman in the picture is an actress who is performing”), they use top-down cognitive regulation of their emotion that recruits the cognitive control. To deal with emotions, individuals employ a wide variety of emotion-regulatory strategies, ranging from altering the trajectory of an unfolding emotional response by mentally transforming, to reappraising the meaning of the emotion-eliciting situation [10, 28]. For the reappraisal, the instruction provided along with the experimental stimulus actively elicits explicit emotion regulation and changes the way the subject appraises the meaning of an emotional stimulus. This task is followed by what we term a “modulated recovery period” from the emotional response since the instruction necessitates that the participants change their emotion when confronted with the emotional stimulus. If no such instruction is provided, the subsequent recovery is instead regarded as a natural recovery period achieved by people themselves, which is named as the implicit regulation. Gyurak et al. further defined the implicit regulation of emotion as that individuals are (i) unaware of the modulation of emotional control induced by the stimuli on their behavior and (ii) the regulatory process is implemented largely outside of awareness [24]. According to the definition, one emotion regulation process without instructions for drawing attention to control the emotional state can be recognized as implicit, even though other mental processes are required. For example, Etkin and colleagues adopted the emotional conflict task (naming facial expressions vs. reading semantic labels written over the expressions) based on the classic Stroop paradigm to realize the implicit emotion regulation by trial-to-trial changes in the congruency effect [29, 30]. The conflict on trial *n* − 1 triggers an increase in emotional control, thereby reducing susceptibility to emotional conflict on trial *n* unconsciously. Nevertheless, complicated task commands can also make participants nervous, and distract them from the natural emotional baseline, although no explicit instruction is conveyed to ask them to notice or change their emotions. By contrast, Yang and colleagues investigated the emotional self-regulation during a natural recovery period following the discomfort induced by viewing aversive pictures (with fearful and disgusting contents) [31]. Only “viewing picture” and “taking a rest” (after viewing all pictures) were cued to the participants in the experiment. Based on previous studies reporting the habitual use of emotion regulation [32, 33], individuals ‘‘intuitively’’ regulate emotion and quickly decrease negative affect in demanding situations to facilitate higher order goal pursuit, regardless whether they are explicitly prompted to do so. This habitual tendency to spontaneously respond to stress or negative emotions was supported by Yang’s study. They identified the concurrency of the increasing activation in the dorsolateral prefrontal cortex (DLPFC) and decreasing activation in the basal ganglia during a “non-directive” recovery, which verified the involvement of cognitive regulation in even emotional self-recovery without explicit consciousness. However, relative to the stress response or emotional contagion, researches on the natural self-recovery from negative emotion is still insufficient. In reality, willfully and consciously employed emotion-regulatory strategies can reliably alter the course of emotional responding, but it is also apparent that people do not and will not pursue conscious regulatory goals unless they have the motivation and the ability to do so. Therefore, it is valuable to work out the mechanism of natural recovery in detail.

Concerning on how emotions can be modulated, previous studies have proposed several classic cognitive models, most of which were proposed to conceptualize the procedure of explicit emotion regulation. Gross proposed the emotion generative process going through five stages, including selection of the situation, modification of the situation, deployment of attention, change of cognitions, and modulation of responses [34]. Combined with the neuroimaging researches, the five-stage process deploy multiple brain regions covering the frontal, parietal, temporal cortices, and subcortical areas to cooperate and achieve emotion regulation [23, 34]. Wager et al. explicitly focused on cortical–subcortical interactions to elucidate the regulatory processes underlying successful emotion regulation, and highlighted the critical role of the ventrolateral prefrontal region (VLPFC) in both the generation and regulation of emotion [35]. Ochsner et al. proposed a model that specifies how prefrontal and cingulate control systems modulate activity in perceptual, semantic, and affect systems as a function of one’s regulatory goals, tactics, and the nature of the stimuli and emotions being regulated [27]. Most importantly, they found that DLPFC can directly regulate the brain activation of ventral striatum (VS) and amygdala as well as the subjective emotional experience of individuals [36]. Furthermore, Kohn et al. proposed a 3-stage working model aiming to integrate the modal model of emotion regulation and appraisalist theories of emotion [23]. These models are presented at different time, and the later models inevitably took in the advantages of the earlier models. Therefore, commonalities can be found in them, such as the core brain systems and basic steps involved in the regulatory processes. But a full consensus has not yet been achieved. For instance, it is still unclear whether VLPFC involved in both emotion regulation and emotion generation; Whether DLPFC can directly act on VS or amygdala to achieve a direct emotion regulation pathway. On the other hand, a complete understanding of emotion regulation and its role in mental health not only relies on in-depth explorations of the explicit form, but also requires further investigations of implicit forms of regulation. The dual-process framework elaborated that both the explicit and implicit forms are the two sides of emotion regulation, and not mutually exclusive categories [24], which has been supported by the overlapping processes revealed in neuroimaging studies [37, 38]. Thus, we speculate that the cognitive models of emotion regulation mentioned above also apply to the implicit emotion regulation to some extent.

To solve above problems, the dynamic causal modeling (DCM) was utilized in this study to unveil the effective connectivity in the brain network underpinning the implicit emotion regulation, which is an adequate approach to model how the directed neuronal connectivity generates the observed blood oxygenation level-dependent (BOLD) signal during the emotional activity [39]. The fMRI data in Yang’s study on emotional self-recovery was reanalyzed to examine the architecture comprising four key brain regions with high repetition across the emotion regulation models [31]. Dynamic causal models were constructed to figure out how the relevant brain regions communicate to promote the natural shift of mental state in response to the aversive emotion; and which model best described the data.

## Materials and methods

### Participants

Twenty right-handed healthy postgraduate students (10 females) were recruited in the previous fMRI experiment, with the mean age of 25 ± 1.3 years and normal or corrected-to-normal vision [31]. Their data were re-used for the construction of dynamic causal models. None of them reported any history of neurological or psychiatric diseases. During the data analysis, one subject’s data was excluded because his brain activation induced by the task condition was too weak to extract the time series based on the unified MNI coordinates across all the participants. Data from the remaining 19 participants were used for the following analysis. All the participants signed the informed consent and this study was approved by the Ethics committee of Xuanwu Hospital, Capital Medical University.

### Stimuli

The emotional self-recovery experiment applied 15 aversive pictures and 15 neutral pictures to visually induce the aversive emotion of each subject. The pictures were selected from the International Affective Picture System (IAPS) based on normative ratings in valence and arousal. The contents of pictures involve snakes, spiders, attacks, bloody wounds, and dead bodies, with the mean valence of 2.61 ± 1.60 and the mean arousal of 6.30 ± 2.14. In contrast, pictures of household items against simple backgrounds (e.g., a cup on a table) were used as the neutral stimuli, with the mean valence of 5.01 ± 1.13 and the mean arousal of 3.05 ± 1.94.

### Experimental design

A within-subject design with “pre–post” tests was utilized to implement the fMRI experiment. We presented the neutral pictures only in the pre-test session, and aversive pictures only in the post-test session. In each session, 15 emotional pictures were successively displayed for 1 min, at a rate of 4 s per picture. Participants were required to view all the pictures carefully, without any instruction on taking actions to cope with the evoked emotion. As the natural recovery phase from the aversive stimuli, a 4-min resting period subsequently followed the picture viewing stage, in which participants were asked to keep their eyes open and look at a white “+” sign against the black background of screen. However, different from the previous study, the present investigation only picked the 1st min of the participants’ data as the research window. Since we believe that participants continuously concentrated on the aversive pictures, so that it was not easy for other mental activities to enter participants’ mind during the 1 min, which makes the period excellent for exploring the emotion-related processes. Meanwhile, previous studies indicated that one’s habitual tendency might predispose individuals to quickly and effectively deal with their unfolding emotions when they confront stress or negative emotions, even if the emotions are triggered subliminally [40]. So the emotion regulation occurred just behind the emotion generation, and both of which were included in the 1-min period; the participants went through a natural and self-emotional recovery simultaneously when they viewed the pictures. The experimental design is illustrated in Additional file 1: Figure S1.

In our previous study, it was found that the caudate nucleus as an important component of the striatum was activated when individuals were engaged in emotional self-recovery, which not only participated in the generation of emotions, but also played a role in the suppression of aversive emotion. Based on this finding and the 3-stage working model proposed by Kohn et al. [23], we heuristically divided the 1-min period for viewing pictures into three stages: 1st–10th seconds, 11th–30th seconds, and 31st–60th seconds, corresponding to the three stages of perceiving the stimulus and initially generating emotion (perception), suppressing emotion (inhibition), and spontaneous cognitive regulation (modulation), respectively. The reasonability of the division of stages will be deliberated in Sect. 4.

### MR data acquisition

A 3.0-T MRI system (Siemens Trio Tim; Siemens Medical System, Erlanger, Germany) and a 12-channel phased array head coil were employed for the scanning. Foam padding and headphone were used to limit head motion and reduce scanning noise. 192 slices of structural images with a thickness of 1 mm were acquired by using a T1-weighted 3D MPRAGE sequence (TR = 1600 ms, TE = 3.28 ms, TI = 800 ms, FOV = 256 × 256 mm^2^, flip angle = 9°, voxel size = 1 × 1 × 1 mm^3^). Functional images were collected through a T2 gradient-echo EPI sequence (TR = 2000 ms, TE = 31 ms, flip angle = 90°, FOV = 240 × 240 mm^2^, matrix size = 64 × 64). Thirty axial slices with a thickness of 4 mm and an inter-slice gap of 0.8 mm were acquired.

### Data preprocessing

The SPM12 software (Wellcome Trust Centre for Neuroimaging, London, UK, http://www.fil.ion.ucl.ac.uk) was applied for the fMRI data preprocessing. The first two images were discarded to allow the dynamic equilibrium of magnetization and the remaining 150 volumes (5 min) were kept for the following analyses. The functional images were corrected with the slice-timing differences and reallocates the median image to correct the rigid body motion. No Case excessed the head motion criteria (greater than 2 mm or 2°). The high-resolution anatomical images were co-registered with the average images of EPI series, and then the space was normalized to the MNI template. After applying spatial normalized parameters to EPI images, all volumes were resampled to 3 × 3 × 3 mm^3^, and 8 mm FWHM isotropic Gaussian kernel was adopted for smoothing to keep consistency with other task-based experimental data.

### SPM univariate analysis

Data from the two sessions (pre-test and post-test) were statistically analyzed by using SPM12. In one session, images of the three stages (perception, inhibition, and modulation) were created individually based on the general linear model. In the group-level, paired *t*-tests were implemented for each stage to examine the different activation patterns between the pre-test and post-test, followed by one-sample *t*-tests performed for the contrast images. Activations reported survived an uncorrected voxel-level intensity threshold of *p* < 0.001 with minimum cluster size of *k* > 30 voxels.

### Dynamic causal modeling

DCM is a biophysical model that describes the underlying neuronal connectivity and how that connectivity generates bold signals that are observed. In short, the priori selected mathematical model of the underlying neuronal connectivity between a set of brain regions (nodes) is a bilinear differential equation of state system, with coefficients specified by three matrices (A matrix, B matrix and C matrix) [39].

DCM analysis can identify which specific nodes in the model present effective (directional) connections to other specific nodes in the model, which nodes receive the model’s driver input from experimental conditions, and which connections between specific nodes in the model are modulated under experimental conditions. Brain regions in the model that first experience changes in neuronal activity related to experimental conditions are defied as nodes receiving drive inputs, and quantified by C matrix parameters. Nodes that receive driver input then influence (“driver”) connections to other nodes in the model. The endogenous (or fixed) connectivity in DCM, i.e., the infrastructure of the network is quantified by A matrix parameter that measures the effective connectivity strength (in Hz) between nodes, regardless of the input transient switch. Experimental conditions can regulate the endogenous connectivity between nodes. These modulation effects are quantified by B matrix parameters that increase or decrease the connection strength compared to the endogenous connectivity at different times in the experiment, which are time-dependent changes under specific experimental conditions [41].

### Regions of interest and time series extraction

Constructing a DCM follows three steps: (i) specifying a design matrix for external or experimental input; (ii) extracting time series stored in volumes of interest (VOIs) file; (iii) indicating on the adjacency matrix or graph which connections are present and how they are influenced by experimental conditions. Brain regions showing effects related to perception, inhibition, and modulation were identified using the group-level random effects in the aforementioned SPM univariate analysis. Among them, four regions that frequently reported as key nodes for facilitating emotion regulation in the classic theories were selected to constitute the DCMs, including the dorsal portion of anterior cingulate cortex (dACC), dorsolateral prefrontal cortex (DLPFC), ventrolateral prefrontal cortex (VLPFC), and ventral striatum (VS) (shown in Additional file 1: Figure S2). Time series were extracted from the four nodes in the form of VOIs, based on their MNI coordinates showing the greatest *T*-scores in the univariate analysis. The same VOIs were applied to each subject. The number of voxels and center of mass of the four VOIs are shown in Table 1.

**Table 1:** Number of voxels and the MNI coordinates of the four VOIs used as nodes in the DCM analysis

VOI	Number of voxels	MNI coordinates [*x*, *y*, *z*]
R. dACC	31	6, 15, 33
L. DLPFC	45	− 27, 30, 33
L. VLPFC	67	− 24, 54, − 6
L. VS	121	− 30, − 15, 0

*dACC* dorsal portion of anterior cingulate cortex, *DLPFC* dorsolateral prefrontal cortex, *VLPFC* ventrolateral prefrontal cortex, *VS* ventral striatum, *L* left, *R* right

### DCMs specification and model comparison

As implemented in DCM 12 in SPM12 (http://www.fil.ion.ucl.ac.uk/spm), we then specified 50 pre-defined DCMs with the four selected nodes (VOIs) based on classic theories of emotion regulation in addition to our new hypotheses, by setting discrepant patterns for the within-node (intrinsic) and between-node (extrinsic) connections, as well as for both the endogenous (fixed) connectivity and modulation effects. The main differences between models are, whether an endogenous connection between two specific nodes exist or not; whether an endogenous connection between two specific nodes was unidirectional or bidirectional; whether the regions for receiving driver input are the same or not, and so on. The diagrams for illustrating the 50 DCMs are exhibited in Additional file 1: Figure S3. The term “modulation effect” denotes bilinear modulation effects in the context of a deterministic DCM. This driver input is modeled using a C matrix. The rest of the input only moderates the internal and external connections. These modulation effects were modeled using two B matrices.

With the help of Bayesian model selection (BMS) family-level inference, DCM allows for quantitatively comparing which model architecture explains the observed data best [42]. Furthermore, the posterior probability of the model parameters and their mean values across different participants can be calculated by using Bayesian model averaging (BMA), and can thus elucidate the connectivity changes within a network during specific task conditions.

Afterward, a fixed effect (FFX) approach across performed experimental stages and a random effect (RFX) approach to account for inter-subject variability were conducted to generalize the results of these analysis to the population, and to work out the final parameters. With respect to the modulatory parameters of the optimal model, positive parameter values indicate that increasing activity in a region results in increasing rate of change in the connected region, whereas negative parameters indicate that increasing activity in a region results in decreasing rate of change in the connected region [41].

## Results

### Differences in brain activation between pre- and post-tests

**Fig. 1:**
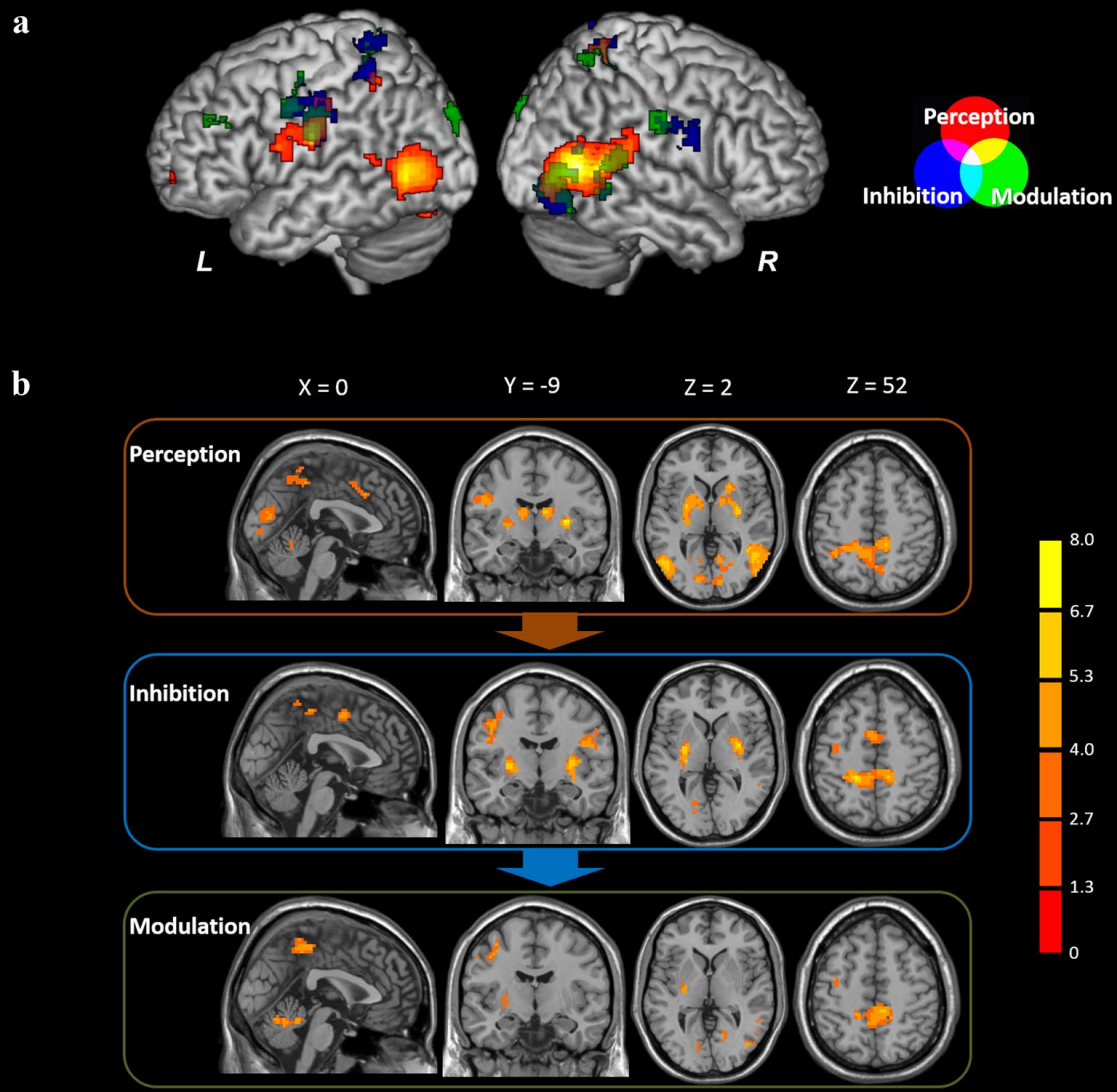
Images were compared in couples between the two sessions for each stage with the paired *t*-tests. Increased activation was identified only in the contrast of post-test > pre-test with the threshold of *p* < 0.001 (uncorrected) and *k* > 30, revealed in MNI coordinates. The color bar indicates the *t*-score. **a** Brain activations specific to the three stages (perception, inhibition, and modulation) are overlapped and presented on the rendered human brain model. **b** Brain activations are shown for each stage using the three-view drawing

Paired *t*-tests were performed between the pre- and post-tests. The results showed that the aversive images triggered stronger and broader activation in the post-test, while the neutral images only activated the visual cortex in the pre-test. During the 1-min picture viewing, images were contrasted in couples between the two sessions for each stage, including the perception, inhibition, and modulation stages. Increased activation was identified only in the contrast of post-test > pre-test, which may be associated with the discomfort caused by the aversive stimuli (shown in Table 2 and Fig. 1). In the perception stage, significant activations in the brain regions were observed in visual cortex, bilateral striatum (including bilateral putamen and right caudate nucleus), left ventrolateral prefrontal cortex (VLPFC), superior temporal gyrus, postcentral gyrus, precentral gyrus, precuneus, and right middle temporal gyrus, cingulate gyrus. In the inhibition stage, significant activations were found in the brain regions similar to those identified in the perception stage, only except the left VLPFC. In the modulation stage, the pattern of brain activation was almost consistent with the previous two stages, with the absence of the left VLPFC and left/right cingulate cortex, and the new attendance of the left dorsolateral prefrontal cortex (DLPFC).

**Table 2:** Activated regions revealed by the paired *t*-tests

Stage	Region	BA	Cluster	Peak MNI coordinates			*T*-score
				*x*	*y*	*z*	
Perception	R. Cuneus	7	264	18	− 60	24	7.36
	R. lingual gyrus	19					
	L. fusiform gyrus	19	40	− 26	70	− 6	4.75
	R. putamen		69	30	− 9	6	6.40
	R. caudate nucleus		44	32	16	42	4.80
	L. putamen		86	− 27 to 3	− 3	3	6.29
	L. VLPFC	10	37	− 24	54	− 6	4.96
	R. MTG	22	416	45	− 60	9	8.68
	L. STG	22	48	− 51	− 45	15	4.46
	L. postcentral gyrus	3	113	− 57	− 15	27	6.71
	L. precentral gyrus	6	96				
	R. cingulate gyrus	32	54	6	15	33	5.10
	L. precuneus	18	140	12	− 39	48	6.11
Inhibition	R. ITG	19	61	42	− 54	− 12	6.07
	R. MTG	37	58	42	− 48	6	5.44
	L. STG	41	30	− 39	− 39	15	6.43
	R. cuneus	18	44	18	− 75	15	4.38
	L. lingual gyrus	18	38	− 15	− 78	12	4.21
	L. precuneus	7	117	− 18	− 39	54	7.23
	R. putamen		60	33	− 6	3	6.39
	L. putamen		52	− 30	− 12	3	6.30
	R. precentral gyrus	6	58	45	− 12	24	5.99
	L. precentral gyrus	6	159	− 42	− 18	39	6.55
	L. cingulate gyrus	24	34	− 6	0	48	4.92
Modulation	L. DLPFC	9	53	− 27	30	33	4.95
	R. postcentral gyrus		32	54	-18	36	5.01
	L. postcentral gyrus		31	− 63	− 15	33	6.43
	L. precentral gyrus	6	42	− 54	− 3	42	5.04
	L. putamen		30	− 30	− 15	0	6.62
	R. ITG	19	38	42	− 51	− 12	5.20
	R. STG	22	59	45	− 48	12	4.94
	R. precuneus		117	6	− 36	51	6.02
	R. cuneus	18	61	15	− 81	15	5.06
	L. cuneus	18	48	− 9	− 81	12	4.72

All regions survived the statistical threshold of *p* < 0.001 (uncorrected), cluster size *k* > 30 voxels

*VLPFC* ventrolateral prefrontal cortex, *MTG* middle temporal gyrus, *STG* superior temporal gyrus, *ITG* inferior temporal gyrus, *DLPFC* dorsolateral prefrontal cortex, *L* left, *R* right

### Results of DCM analysis

**Fig. 2:**
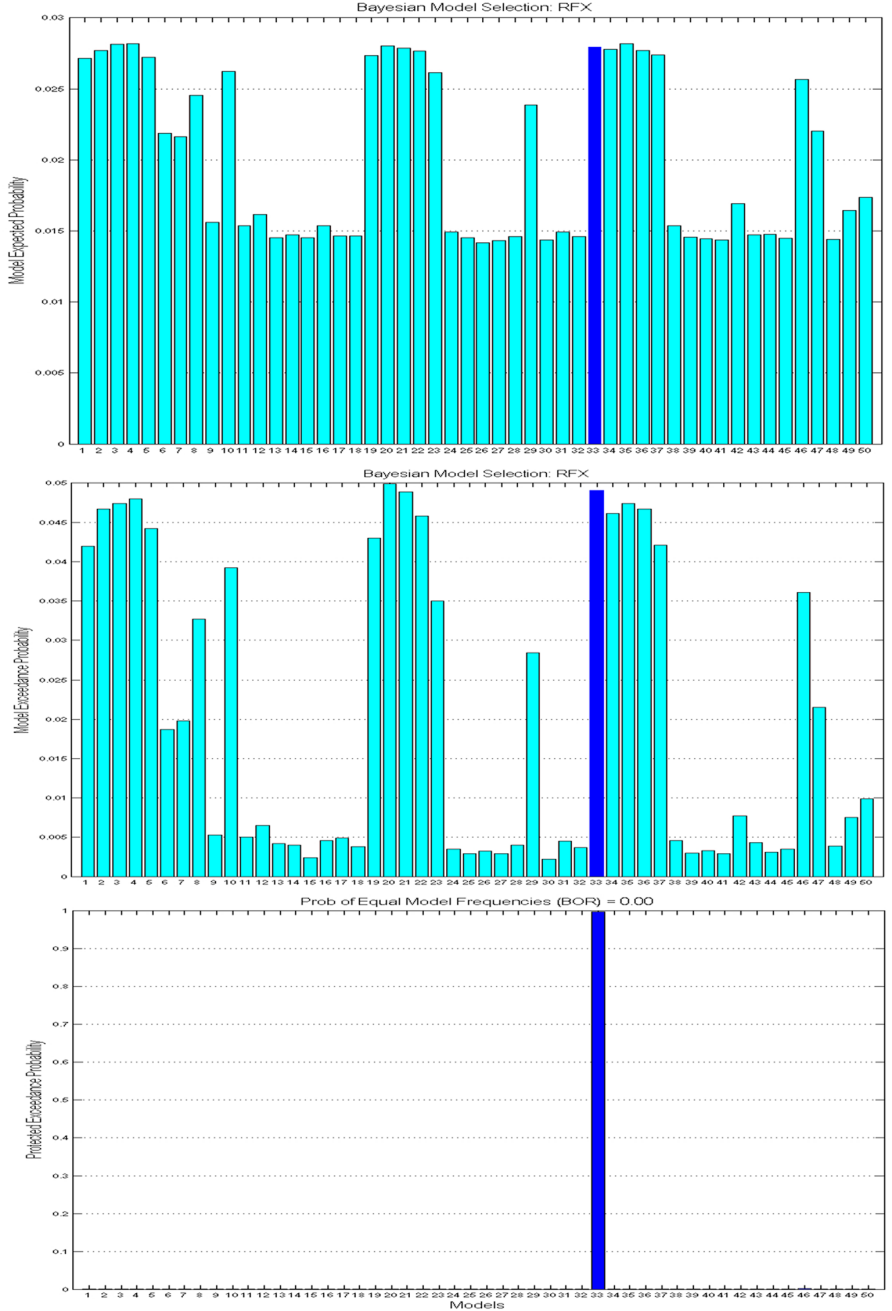
Results of the model comparison revealed by the Bayesian model selection, over the 50 pre-defined models of emotion regulation constructed on the basis of classic theories or the new hypotheses. The 33rd model won the comparison, and was recognized as the optimal model

**Fig. 3:**
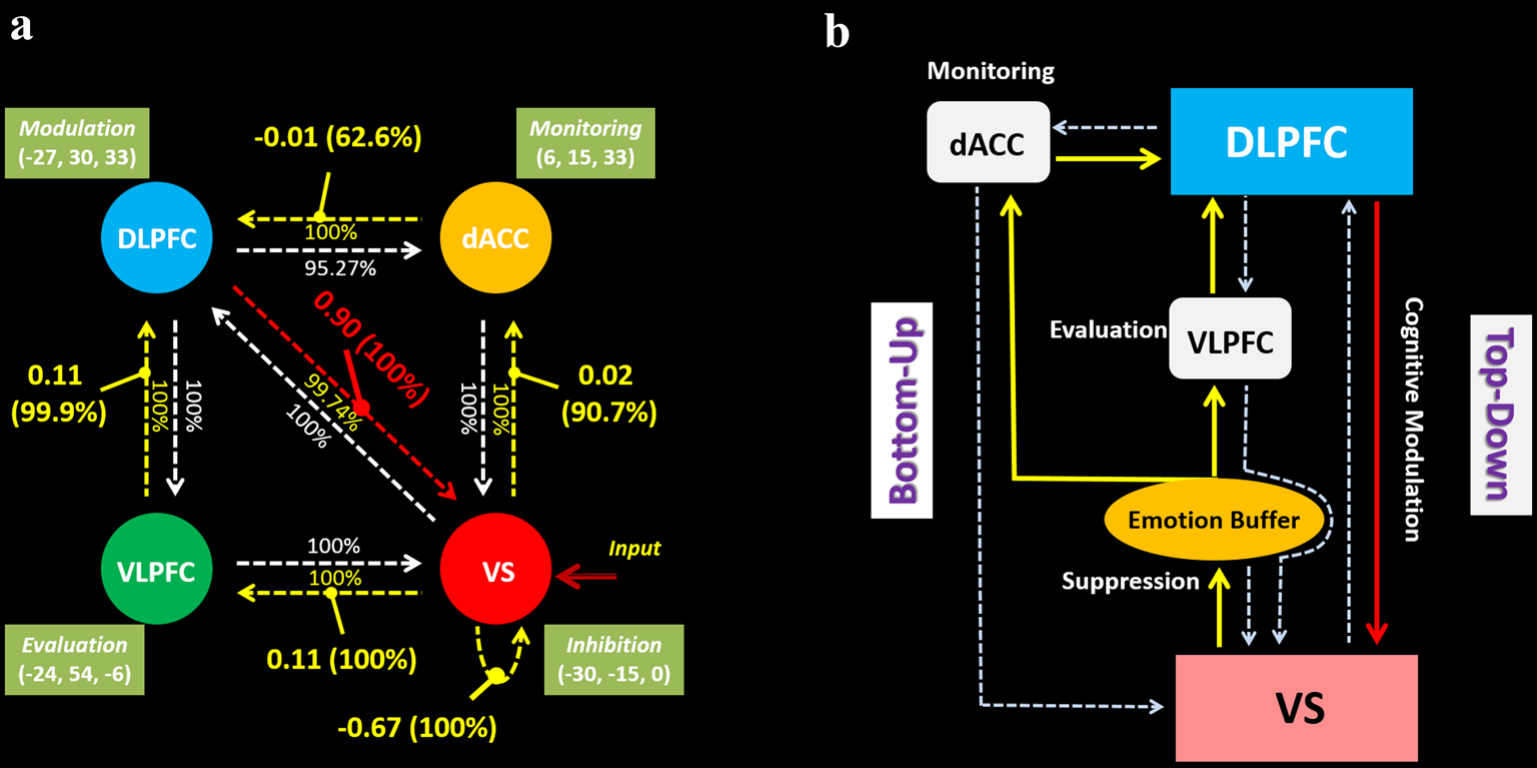
Dynamic models of implicit self-regulation on aversive emotions. **a** The No. 33 model was identified as the optimal dynamic causal model among 50 hypothesized models. The dotted lines between any pair of nodes denote the endogenous connections. The dotted lines in yellow or red mean that the corresponding endogenous connections receive modulatory effects during specific task conditions; the dotted lines in white stands for endogenous connections without being modulated; the solid but short lines denote the modulations imposed on endogenous connections by task conditions; the red arrow means the driver input. Bidirectional endogenous connections were identified between each pair of nodes, except the one between dACC and VLPFC. Connection strength and posterior probability are reported for each modulatory effect. **b** The optimal model is abstracted as a dual regulatory model that is proposed for the implicit self-regulation of aversive emotions. The bottom-up regulation involves an indirect pathway initiated from the VS to the DLPFC via the VLPFC and dACC, respectively, while the top-down regulation involves a modulation imposed by the DLPFC on the VS directly. *dACC* dorsal portion of anterior cingulate cortex, *DLPFC* dorsolateral prefrontal cortex, *VLPFC* ventrolateral prefrontal cortex, *VS* ventral striatum

50 distinct DCMs established using the same four brain regions (VS, VLPFC, DLPFC, and dACC) were compared to find the optimal sparse model structures over participants. Models with redundant connections (and modulations) showed lower posterior probability and lower possibility to be reserved. Finally, the 33rd model showing the highest posterior probability (99.75%) won from the comparison (shown in Fig. 2). The connectivity matrix (A matrix) in the optimal model exhibited a highly interconnected (dense) infrastructure (shown in Fig. 3a). All the four nodes were linked by bidirectional connections, except the pair between the VLPFC and dACC (the posterior probabilities of all the endogenous connections are shown in Table 3). In terms of the modulatory effects, several connections showed enhanced or inhibitory effects to facilitate emotional self-regulation. As shown in Table 4, the manifested modulatory effects (*Strength, Posterior Probability*) contain the ones imposed on the within-node (intrinsic) connection of the VS itself (*− 0.67, 100%*), and on the between-node (extrinsic) connections from VS to VLPFC (*0.11, 100%*), from VS to dACC (*0.02, 90.65%*), from VLPFC to DLPFC (*0.11, 99.83%*), from dACC to DLPFC (*− 0.01, 62.55%*), and from DLPFC to VS (*0.90, 100%*).

**Table 3:** Posterior probabilities of endogenous connections in the optimal four-node DCM

Connection	Posterior probabilities (%)
VS → VS	100
VS → VLPFC	100
VS → dACC	100
VS → DLPFC	100
VLPFC → VLPFC	100
VLPFC → VS	100
VLPFC → DLPFC	100
DLPFC → DLPFC	100
DLPFC → VLPFC	100
DLPFC → dACC	95.27
DLPFC → VS	99.74
dACC → dACC	100
dACC → DLPFC	100
dACC → VS	100

*VS* ventral striatum, *VLPFC* ventrolateral prefrontal cortex, *dACC* dorsal portion of anterior cingulate cortex, *DLPFC* dorsolateral prefrontal cortex

**Table 4:** Posterior probabilities and connection strengths of bilinear modulation effects in the optimal four-node DCM

Connection	Posterior probabilities (%)	Connection strengths (Hz)
VS → VS	100	− 0.67
VS → VLPFC	100	0.11
VS → dACC	90.65	0.02
VLPFC → DLPFC	99.83	0.11
dACC → DLPFC	62.55	− 0.01
DLPFC → VS	100	0.90

*VS* ventral striatum, *VLPFC* ventrolateral prefrontal cortex, *dACC* dorsal portion of anterior cingulate cortex, *DLPFC* dorsolateral prefrontal cortex

## Discussion

In the present study, we reanalyzed the dataset collected in a previous fMRI experiment which was implemented with the purpose of understanding the neural correlates of natural recovery from aversive emotions, i.e., the implicit emotion regulation. In order to figure out the brain architecture that supports the automatic regulation of aversive emotions and the relevant information-processing pathways, the dynamic causal modeling (DCM) approach was employed, to examine the one best fit the fluctuations of bold signals based on real-data analysis, from 50 pre-defined models established in compliance with the classic theories or newly proposed hypotheses (shown in Additional file 1: Figure S3). After the SPM univariate analysis and DCM specification and comparisons, the optimal model of implicit emotion regulation was obtained, as well as the connectivity/modulation parameters revealed by model estimation.

### A distributed brain architecture underlying emotion regulation

A distributed brain architecture was identified to support the complex processes of the unconscious emotion-recovery. Most of the involved brain regions in the distributed brain network were consistent with those reported in previous studies on both explicit and implicit emotion regulation, such as the ventral striatum (VS), ventrolateral prefrontal cortex (VLPFC), dorsolateral prefrontal cortex (DLPFC), dorsal portion of anterior cingulate cortex (dACC), precentral and postcentral gyri, superior and middle gyri (STG and MTG), and visual cortex, etc.

In general, emotions deploy highly evolutionarily conserved subcortical systems, such as the VS, amygdala, and periaqueductal grey (PAG). These regions are considered as core limbic regions, and may extract simple motivational features of a stimulus, and then provide a quick analysis of the affective properties of the stimulus. Among the regions, the amygdala is well known as a primary node specific to perception and encoding of stimuli related to affective goals. By contrast, the ventral striatum that was activated by the emotional pictures in the present study is associated with learning which cues (ranging from social signals, like smiling faces, to actions, to abstract objects) predict rewarding or aversive outcomes [43, 44]. In addition to the emotional function, another main function of the basal ganglia is to control and regulate the activity of the motor and premotor cortical areas, and to promote voluntary movement and manual response inhibition. The striatum is the main input station of the basal ganglia and is considered as a critical area for giving the “stop signal”. This suggests that the striatum participates in active inhibitory control of the primary motor cortex (M1) by suppression [45, 46]. The VLPFC may not necessarily reflect the regulatory process per se, but signals salience and therefore the need to regulate [47, 48]. It integrates affective valuations of specific stimuli sent by the VS or amygdala with inputs from other brain regions, and detects salience from the overall stimuli, and then functions as the core structure for appraisal processes [35]. Revealed by the DCM, the VLPFC may signal the need for regulation via the direct anatomical connections to the DLPFC [49, 50]. The functional features of DLPFC are usually linked with pure cognitive tasks, such as working memory, reasoning, social cognition and general cognition. It is thought to be the central regulatory region of the brain that processes emotions, and has also been proposed to play an important role in the higher order “cold” regulatory processes [51]. The dACC is functionally related to memory tasks, language processing and behavioral inhibition [52]. The dACC plays a crucial role in intentional action control [53]. Previous studies suggest that the dACC is a key area for controlling emotion-related behavior. It is closely implicated in monitoring reward response behavior and punishment avoidance [54]. The STG is functionally characterized by language processing, which reflects its role as a posterior language and higher order visual processing area [23]. It may play a role in the linguistic rendering of social scenes or mental images, and modulate emotional arousal through an effective connection with the VS or amygdala. 

### Processing stages of implicit regulation of emotion

Compared with mood, emotions are quick and fast-changing phenomena. Based on it, emotion regulation in its implicit form has the advantage of being more efficient and effortless than that in explicit form [24]. Under the unconscious state, people generally appear habitual emotion regulation. In our previous results, the existence of the habitual emotion regulation occur jointly with the emotion generation can be proved [31]. In this study, we selected only one part of the original data corresponding to the period of picture viewing for analysis, because the participants were more concentrated to respond to the aversive stimuli and deal with their emotions. As we introduced before, the emotion regulation initiates just behind the emotion generation, and continuously returns feedback to affect the unfolding of emotion [15].

Several classic cognitive models have been proposed to depict the information processing during the explicit emotion regulation, including the five-stage model proposed by Gross [34], Wager’s model that highlighted the critical role of VLPFC in both the generation and regulation of emotion [35], Ochsner’s model that focused on the direct regulation of the DLPFC on the brain activation of VS and amygdala [27], and Kohn’s 3-stage working model that integrated the advantages of the previous models and took the meta-analysis results of ample neuroimaging studies as reference [23]. In Kohn’s model, the processes of emotion regulation are divided into three stages: (i) affective evaluation that actually contains the perceptual encoding of emotional stimulus and the following evaluation; (ii) initiation of regulation that deploy the VLPFC to signal the need for regulation via the dACC and direct anatomical connections to the DLPFC; (iii) and the execution of regulation that in turn influences activity in the VS and amygdala by DLPFC either directly or via the dACC. This model presents a conceptual architecture about the information processing of emotion regulation, which is close to what we have hypothesized, and provides excellent inspiration. However, it is ambiguous if this 3-stage working model is applicable to elaborate the procedure of implicit recovery of aversive emotion.

Based on the existing studies, we hypothesized that the responses of participants during the one-minute period for viewing emotional pictures would show a tendency to initiate from emotional arousal then gradually transform to emotion regulation. By dividing the observation window into three stages, we found that the results of brain activation analysis verified our hypothesis. In the first stage, the brain activation in the visual area and subcortical area (including the caudate nucleus and putamen) were most apparent (see Fig. 1), indicating the initial perception of the emotional stimulus. In the second stage, the emotional processes triggered the brain activation in the precentral gyrus and postcentral gyrus surrounding the Sylvian fissure; the similar pattern of brain activation was observed across the three stages, and might be implicated in the interpretation of scenes in the emotion pictures, in cooperation with the STG that is an important component of the language system [23]. Whereas, the superior portion of the precentral gyrus and postcentral gyrus revealed greater activation only in the second stage, and seemed to be associated with the functions of motor control, such as the motor planning, motor inhibition, etc. [55]. This inhibition was also considered to be related to the inhibition of facial expression [11], and be involved in inhibitory control of emotion [56]. In the third stage, the left DLPFC was significantly activated, suggesting the involvement of the cognitive regulation of emotion. Therefore, the three stages correspond to the emotional perception, inhibition, and modulation, which is defined by the pattern of brain activation.

Etkin et al. suggested that an emotion can be described as a perception–valuation–action (PVA) sequence (the emotional-reactivity PVA sequence), in which input from the external or internal world is perceived, valued and then triggers an action that alters the external or internal world [15]. However, combined with the findings resulted from the current study, we argue a perception–inhibition–valuation–action (PIVA) sequence for implicit regulation of emotion. With respect to which proposed model best fit the collected data specific to the implicit regulation of emotion in the present study, the following part will give an explanation.

### Dynamic causal modeling of self-regulation on aversive emotion

In this study, data corresponding to the picture viewing stage derived from the same group of 19 volunteers were reanalyzed with dynamic causal modeling (DCM) [39]. This approach for analyzing effective connectivity depicts the influence that one neuronal system exerts on another, and evaluates how well a particular model explains the observed data.

In accordance with the findings in the previous study, the present study replicated mental shifts in human brain across three states during picture viewing (Fig. 1), corresponding to initial responses (perception) to aversive pictures that elicited more significant activation in visual regions, suppressing responses (inhibition) that induced more significant activation in ventral striatum (VS) and supplementary motor area (SMA), and modulatory responses (modulation) that led to significant activation in dorsolateral prefrontal cortex (DLPFC). Dynamic causal interconnections were specified to model the three brain state based on time series extracted from four regions that were activated in this study, and are thought to be critical in emotion regulation [23, 27]: ventrolateral prefrontal cortex (VLPFC), dorsal part of anterior cingulate cortex (dACC), VS, and DLPFC. Due to some controversies about the precise functions of the four regions and their interactions (e.g., whether the VLPFC undertakes generation of emotion; whether the DLPFC imposes modulations on the VS in a direct way), 50 models were specified to cover all the hypotheses, and the optimal model which represented the best fit to the data was identified by using the Bayesian Model Selection (BMS) [57].

In consequence, bidirectional endogenous connections were identified between the pairs of VS and VLPFC, VS and dACC, VS and DLPFC, VLPFC and DLPFC, as well as dACC and DLPFC (Fig. 3a). The simple idea was that prefrontal and cingulate systems would support control processes that modulate activity in subcortical systems that generate emotional responses [58]. Although the amygdala is commonly more preferential for emotion generation than the VS, significant activation was not found in amygdala in this study. Therefore, as discovered in the previous study, the VS was connected to both the emotion generation and elementary regulation by suppressing in this study. The emotional stimuli (input) only entered the VS rather than VLPFC, and led to the inhibition (self-connection) in this region; whereas, the induced emotion was so intense that disentangled the inhibition, and accessed to DLPFC in two indirect paths, via the dACC and the VLPFC. Finally, the DLPFC exerted modulatory effects on VS directly to down-regulate the emotion responses. During a series of processes, the VLPFC and dACC played roles in evaluating valence of afferent signals and monitoring conflicts, respectively [27], while the DLPFC might be related to higher order “cold” regulatory processes. Note that these processes are quite possibly utilized spontaneously during the emotional self-regulation.

Based on the three shifting brain states and the optimal dynamic causal model, we abstracted the results and propose that a frontostriatum subserves a dual regulation model for the emotional self-recovery (Fig. 3b), in which both the bottom-up and top-down regulations are involved. The bottom-up regulation is composed of two situations: for attenuate emotions with negative valence, the VS serves as an “emotion buffer” that can bear the emotions by exerting inhibition to a certain extent, till the negative emotions are defused over time; for intense emotions exceeding the magnitude that the VS can bear, the VS will seek help from the DLPFC by transmitting the information about the intense emotions along indirect paths via the VLPFC and dACC. The top-down regulation is achieved by a direct modulation on the VS imposed by the DLPFC.

## Conclusion

Our findings demonstrated that the cognitive regulation was included during the emotional self-recovery under a natural state after aversive stimuli were received. Furthermore, our findings suggest that both the VS-centric bottom-up and the DLPFC-centric top-down regulations are recruited for self-regulation on negative emotions. The DLPFC will exert modulatory influence on the VS only when the VS fails to suppress the induced emotions by self-inhibition. The DCM analysis is an adequate approach for revealing the dynamics of the effective connectivity underlying the brain processes of human cognition and emotion.

## Supplementary information

**Additional file 1.** Supplementary figures.

## Data Availability

The datasets analyzed during the current study are available from the corresponding author on reasonable request.
