# Supplementary material for: A dynamic causal model on self-regulation of aversive emotion
Source: Brain Inform. 2020 Dec 9;7(1):20. doi: 10.1186/s40708-020-00122-0 (PMC7726072; doi:10.1186/s40708-020-00122-0)
Supplement: Supplementary file 1 — Additional file 1. Supplementary figures. [file 40708_2020_122_MOESM1_ESM.docx]

**Supplementary Materials**

**Figure S1**


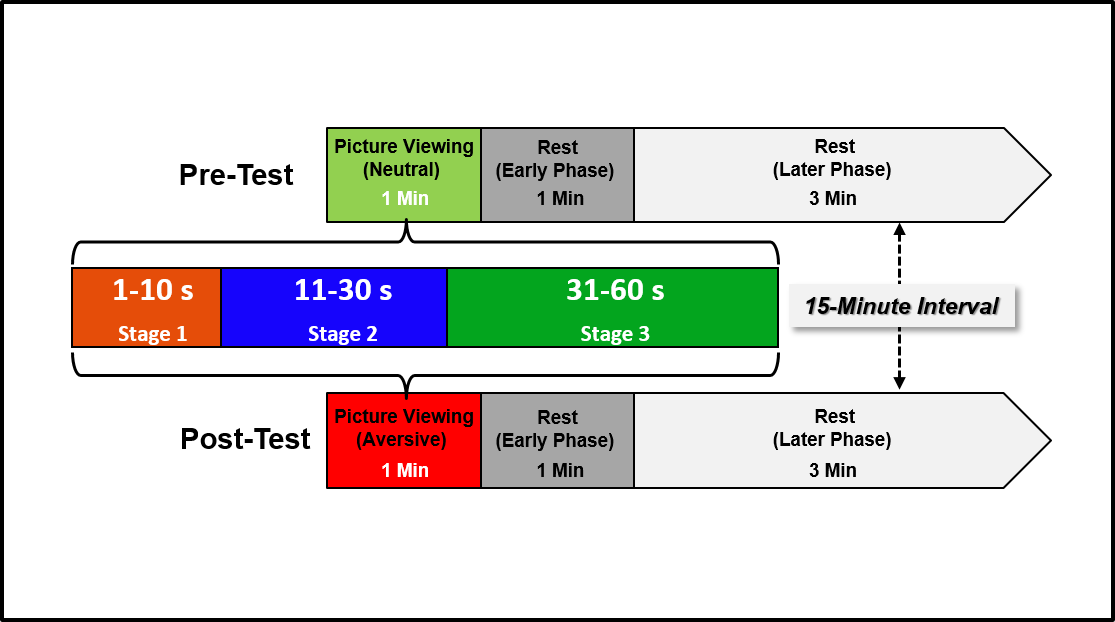


**Fig. S1. Design of the fMRI experiment.** A within-subject design with “pre-post” tests was utilized to reveal the emotional responses and natural recovery induced by aversive pictures. Fifteen aversive pictures were successively displayed for 1 minute, followed by a 4-minute rest. In the present study, only the first 1-minute period was picked, and divided into three stages: 1st-10th seconds, 11th-30th seconds, and 31st-60th seconds, respectively, corresponding to the three stages of perceiving the stimulus and initially generating emotion (perception), suppressing emotion (inhibition), and spontaneous cognitive regulation (modulation).

**Figure S2**


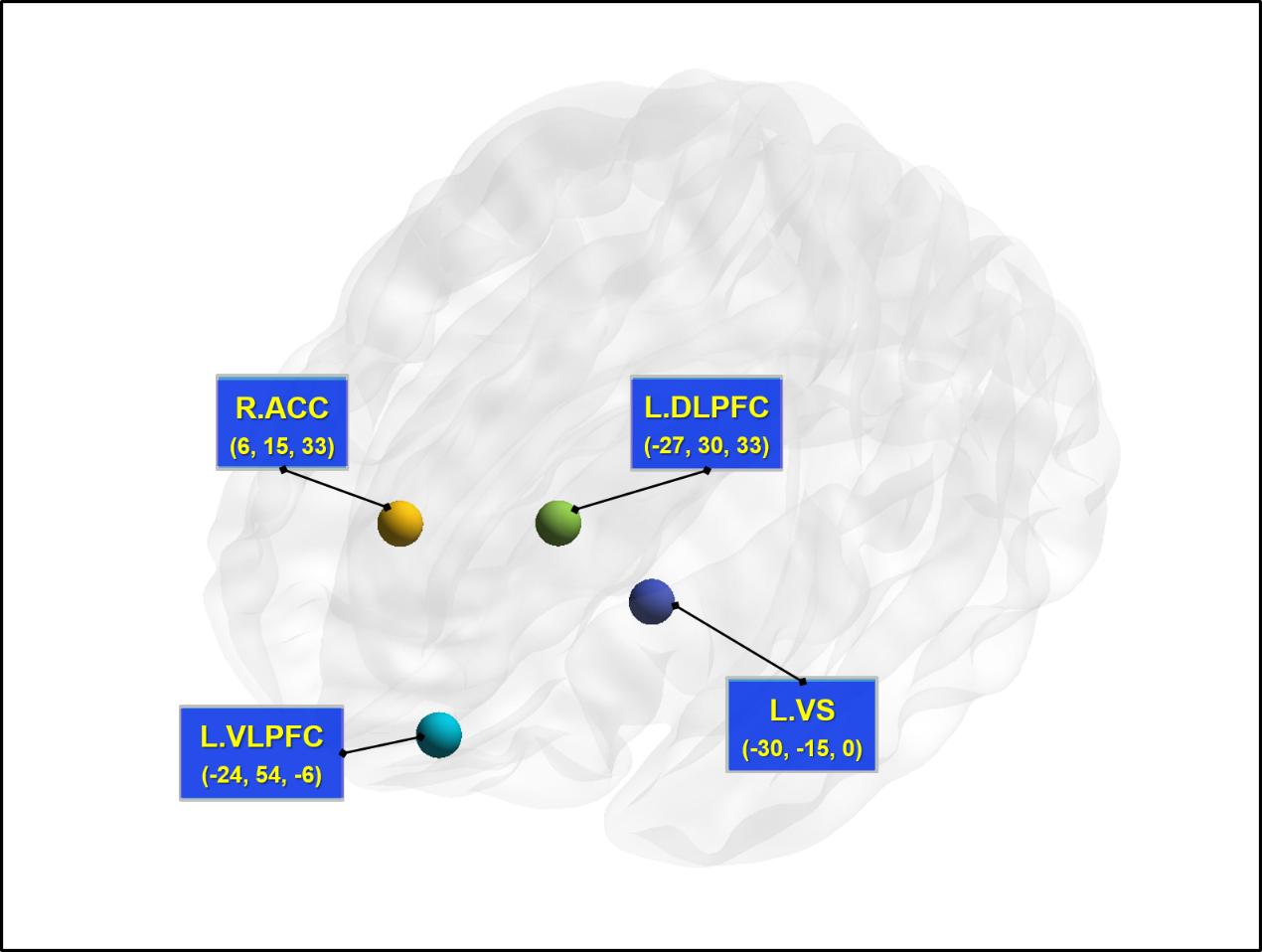


**Fig. S2. Regions of interest and time series extraction for the DCM construction.** Regions or volumes of interest (VOIs) showing effects related to perception, inhibition, and modulation were identified using group-level random effects in the SPM univariate analysis. The VOIs corresponding to four brain regions were selected, including the L. VS, L. VLPFC, R. ACC, and L. DLPFC. The same VOIs (with the same MNI coordinates) were used for each subject for the extraction of time series. Abbreviation: ACC, anterior cingulate cortex; DLPFC, dorsolateral prefrontal cortex; VLPFC, ventrolateral prefrontal cortex; VS, ventral striatum; L, left; R, right.

**Figure S3**


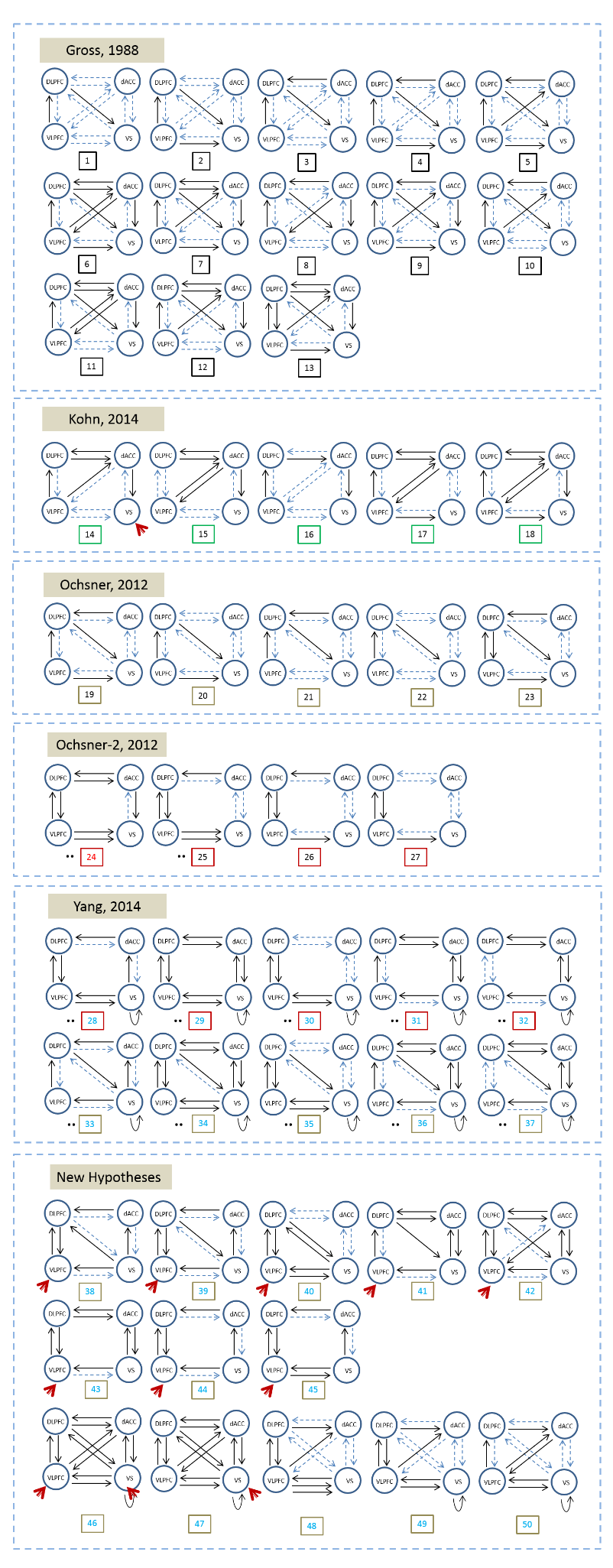


**Fig. S3. Pre-defined fifty DCMs.** Fifty models were established using DCM based on previous classic models of emotional regulation and hypotheses proposed by the authors of the present study. The solid lines denote connections receiving modulatory effects; dotted lines denote endogenous connections without modulation during the experimental conditions; the red arrow denotes the driving input. Abbreviation: dACC, dorsal portion of the anterior cingulate cortex; DLPFC, dorsolateral prefrontal cortex; VLPFC, ventrolateral prefrontal cortex; VS, ventral striatum.
